# Case report: Primary breast osteosarcoma: a case study and literature review

**DOI:** 10.3389/fonc.2024.1433581

**Published:** 2024-12-23

**Authors:** Jingxin Wei, Xiang Luo, Xueping Li, Junqiang Li, Yanling Liang

**Affiliations:** ^1^ Department of Radiology, Guigang City People’s Hospital, Guangxi, China; ^2^ Department of Orthopedics, Guigang City People’s Hospital, Guangxi Medical and 3D Printing Clinical Medicine Research Center, Guangxi, China

**Keywords:** primary, breast, extraskeletal, osteosarcoma, rare

## Abstract

Primary breast osteosarcoma (PBOS) is an extremely rare and poor prognostic malignancy. Here, we presented a 44-year-old female with a primary osteosarcoma of the breast (POB). Comprehensive imaging examinations conducted on the patient can be used to expand the imaging database for cases of breast osteosarcoma.

## 1 Introduction

Osteosarcoma is the most common malignant bone tumor (1), but its incidence is relatively low among malignant tumors, ranging from 1 to 4 cases per million individuals (2). Osteosarcoma predominantly occurs at the metaphysis of long bones (3), with extraskeletal osteosarcoma being less common. Primary breast osteosarcoma is exceptionally rare, accounting for less than 1% of all primary malignant breast tumors (4). To date, there have been fewer than 200 reported cases (5); most are individual case reports or small-scale summaries. There is no consensus on the etiology or radiological characteristics of primary breast osteosarcoma (6). This study reports a case of primary breast osteosarcoma where the comprehensive imaging examinations were conducted on the patient due to an unclear preoperative diagnosis. A literature review is also presented. The results of these imaging examinations can be used to expand the imaging database in cases of breast osteosarcoma.

## 2 Case presentation

**Figure 1:**
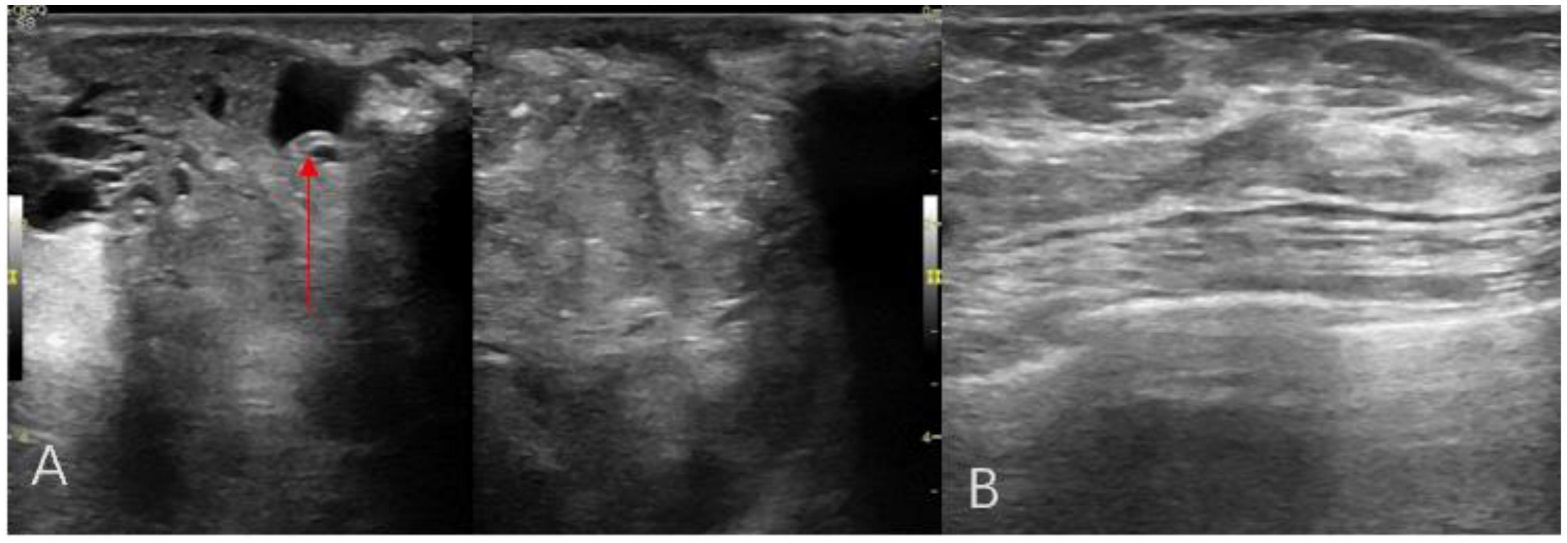
Breast ultrasonography radial scanning **(A, B)** An irregular hypoechoic mass in the right breast, with calcification (red arrow).

**Figure 2:**
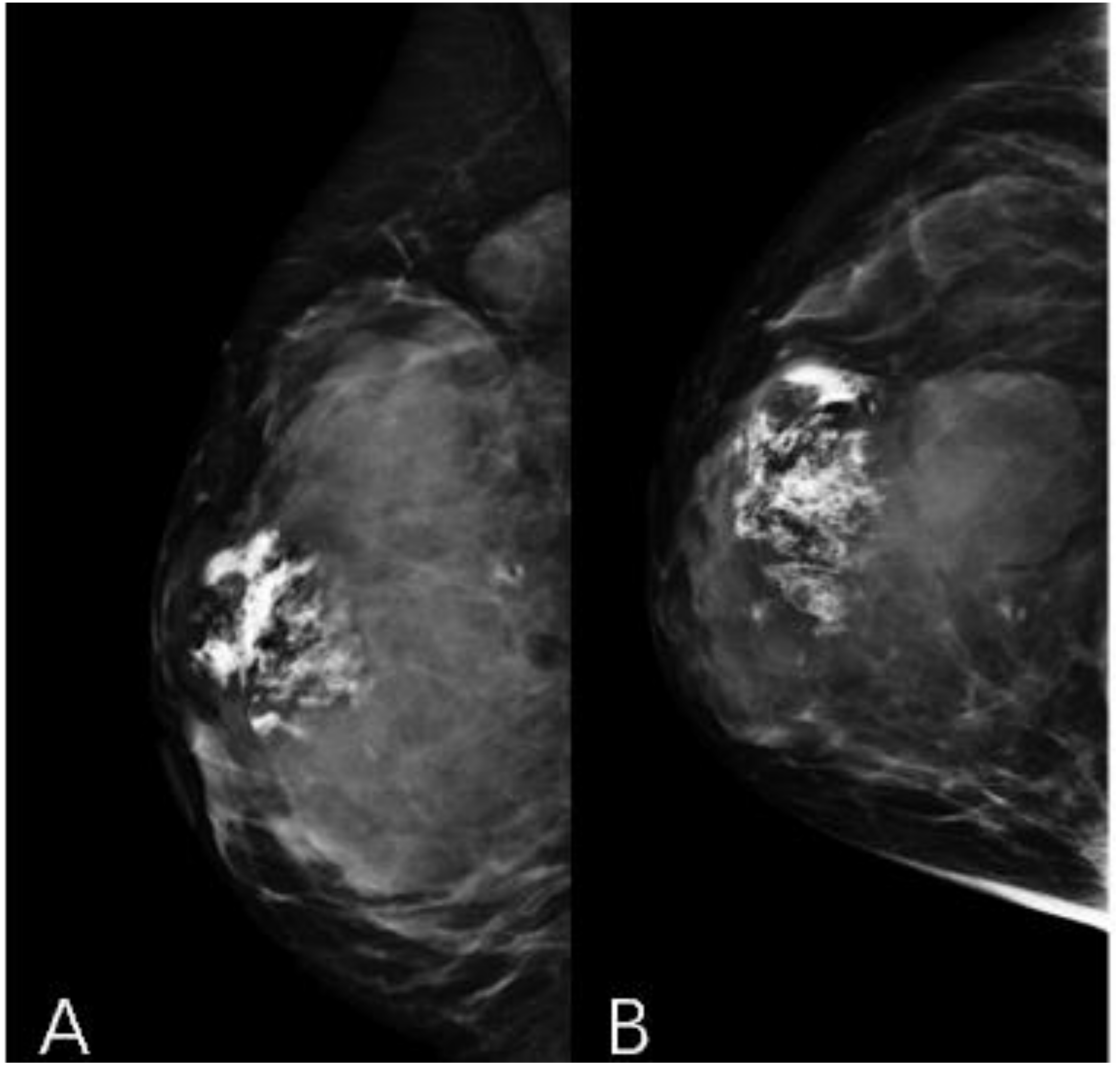
Mammography craniocaudal view **(A)** and mediolateral oblique view **(B)**. A large calcified mass is noted at the right breast.

**Figure 3:**
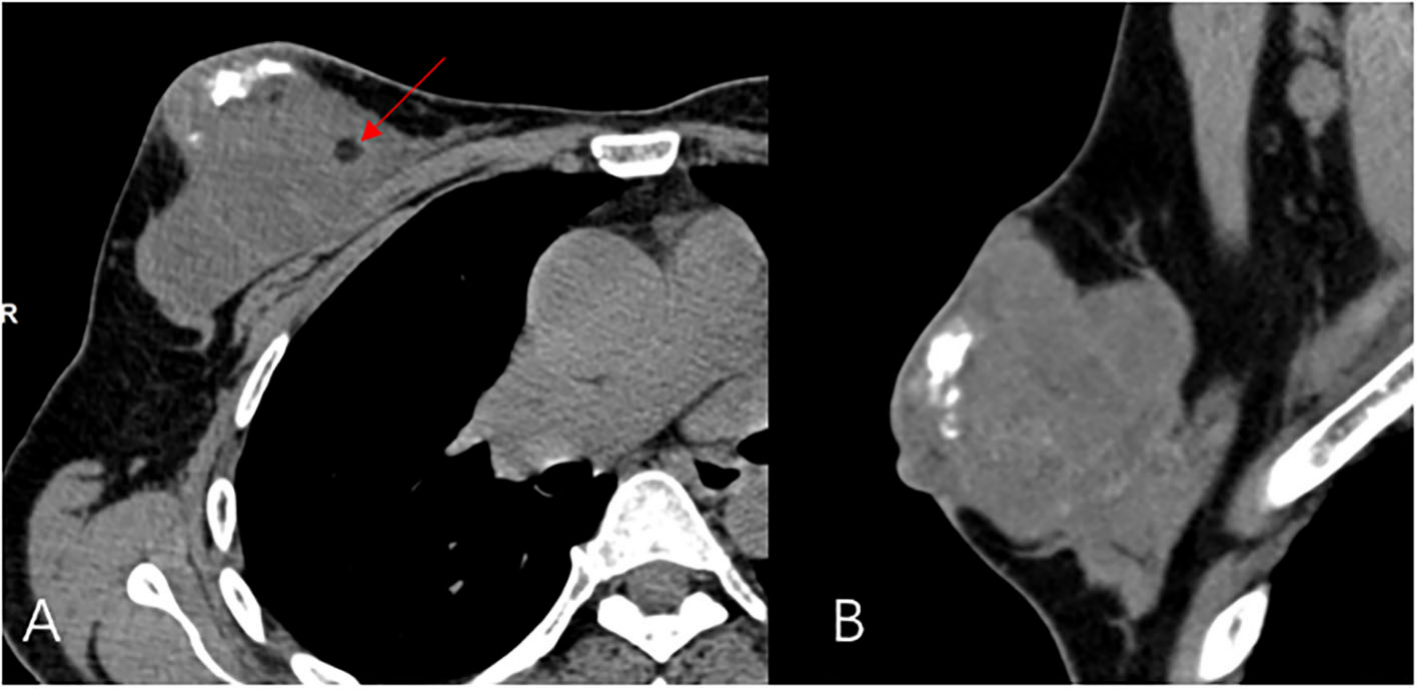
CT Transverse dislocation **(A)** and sagittal **(B)** view, a large calcified mass and round foci of fat (red arrow) is noted at the right breast.

**Figure 4:**
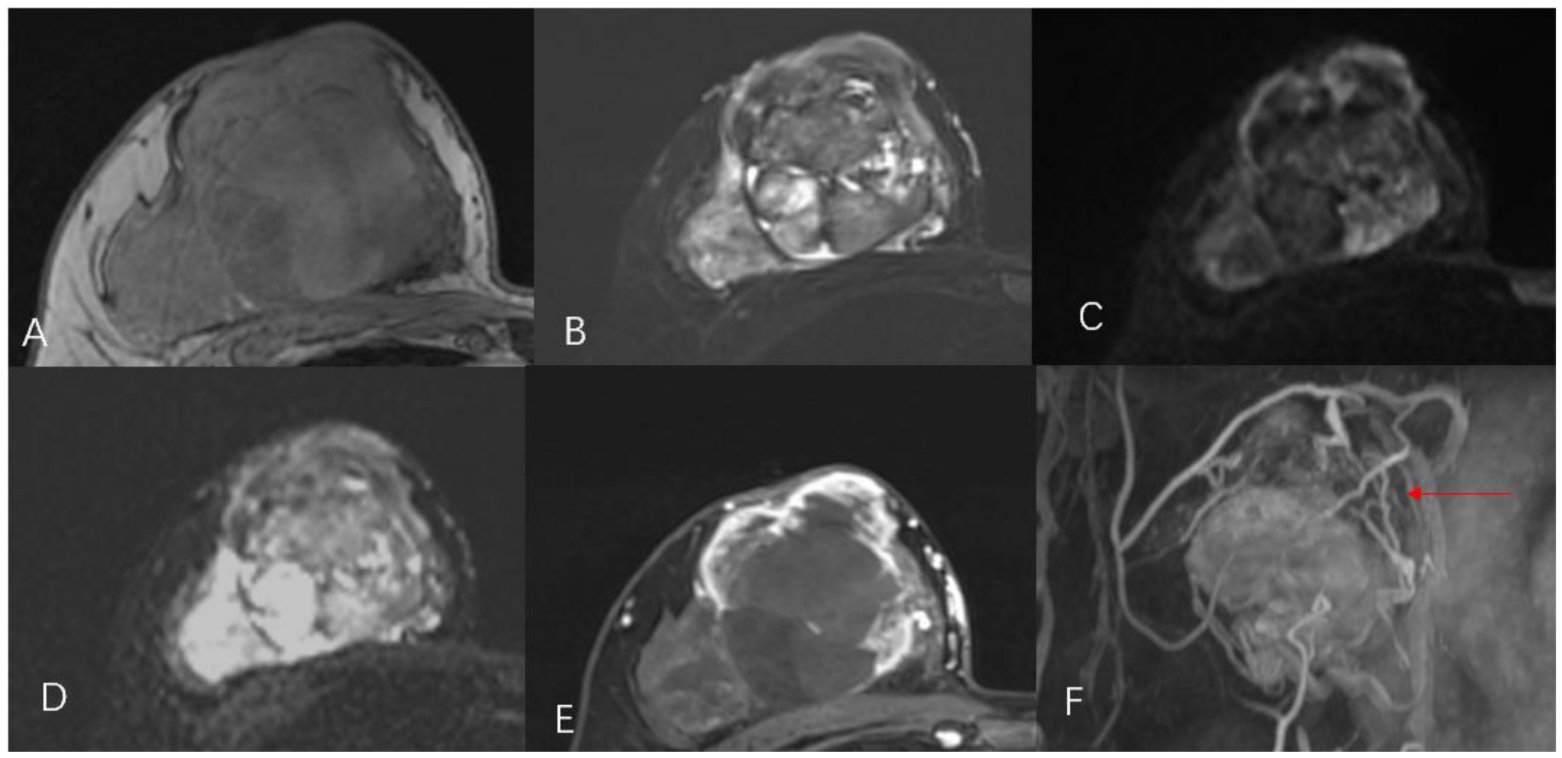
Magnetic resonance imaging T1WI **(A)**, T2WI **(B)**, DWI **(C)**, ADC **(D)**, T1WI with enhancement **(E)**, revascularization **(F)** shows a lobulated mass with numerous large feeding arteries in the periphery (red arrow).

**Figure 5:**
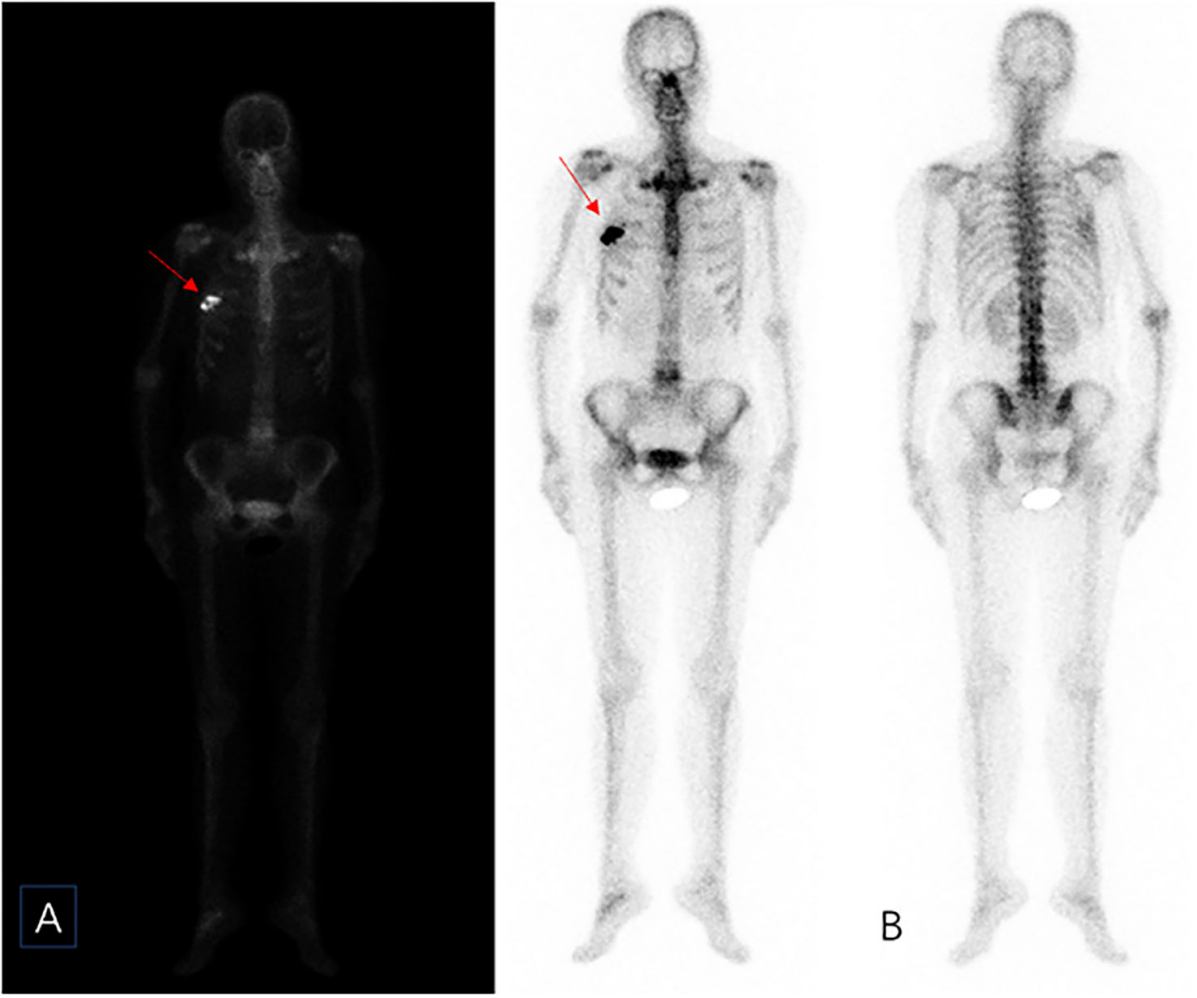
Whole-body bone imaging **(A, B)**. It showed that there was a mass-like abnormal radioactive concentration of outside the bone of the right chest (red arrow), without other abnormal concentrations.

**Figure 6:**
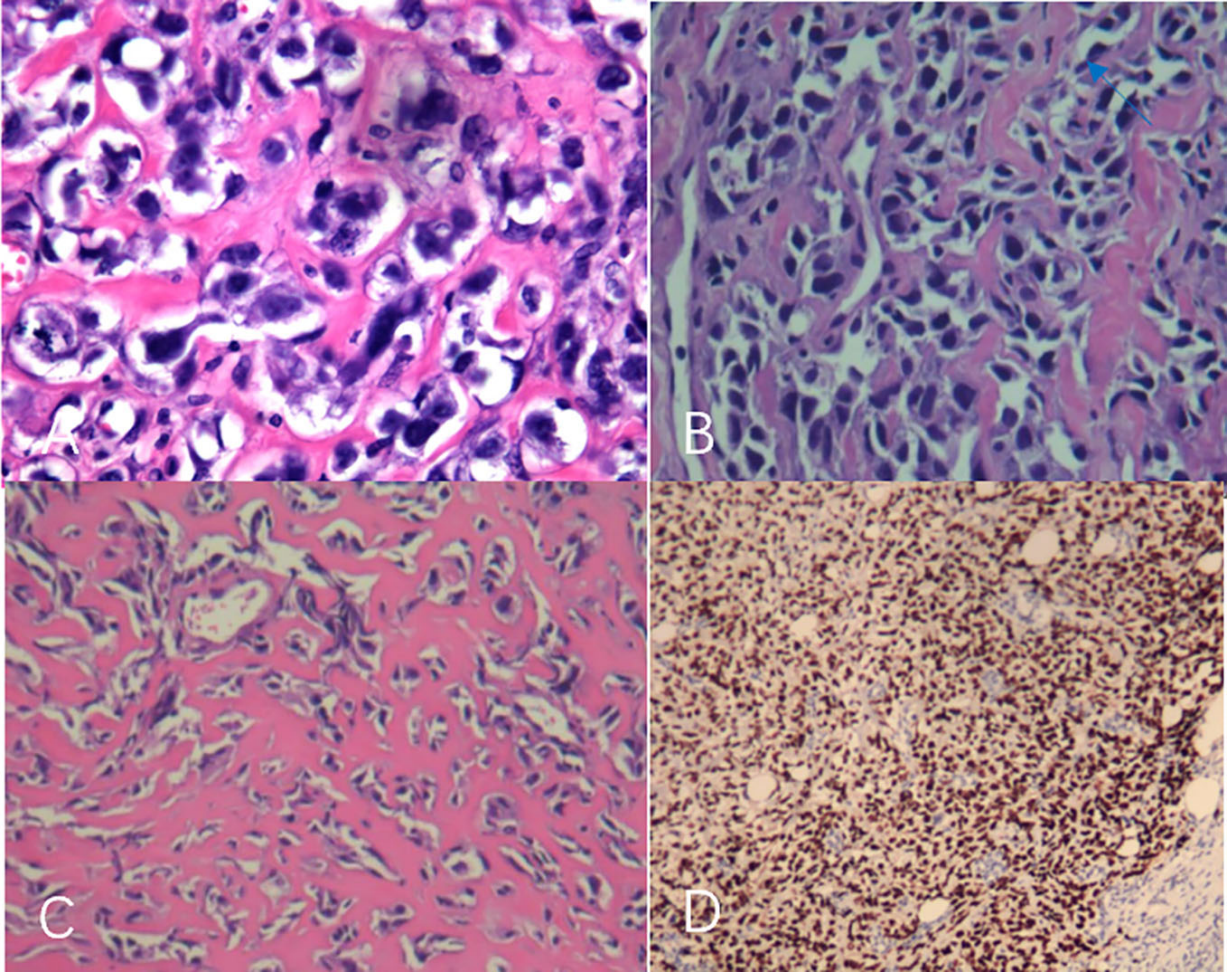
**(A–D)** The tumor was rich in osteoid tissues **(A–C)**, heteromorphic osteoblast-like cells, multinucleated giant cells, and small foci of cartilage, which is consistent with osteosarcoma. Immunohistochemical analysis **(D)**.

A 44-year-old female patient presented to our center with the complaint of a “painless lump in the right breast for the past 2 years.” The patient reported that she incidentally discovered a lump in the upper right breast 2 years ago that was initially the size of a soybean (approximately 1 cm × 0.5 cm) and slightly hard. There was no redness, swelling, warmth, pain, or nipple discharge in the local area. As it did not affect her daily life and work, the patient did not seek medical attention over the past 2 years, during which the lump had progressively increased in size. At the time of the study, the lump had grown to a diameter of approximately 7 cm with slight localized pain. The breasts appeared unequal in size and asymmetrical. The right breast was comparatively enlarged with no signs of redness, swelling, orange peel appearance, or dimpling. The nipple was neither inverted nor deviated, and no discharge was observed. A large lump, measuring approximately 8.0 cm × 6.0 cm, with a hard texture and slight tenderness, was palpable beneath the right nipple. The lump featured a smooth surface, indistinct borders, and a fixed position with poor mobility. The tumor markers: Carcinoembryonic Antigen (CA), CA 125, CA 19-9, and CA 15-3 levels were negative. Subsequently the patient underwent comprehensive imaging examinations including breast ultrasonography (**Figures 1A, B**), mammography (**Figures 2A, B**), and chest Computed Tomography (CT) (**Figures 3A, B**), including breast CT, breast Magnetic resonance imaging (MRI) (**Figures 4A–F**), and whole-body bone Single Photon Emission Computed Tomography (SPECT) (**Figures 5A–C**). The patient underwent complete mastectomy and then adjuvant chemotherapy. Currently, the patient has been living for 2 years without any recurrence. Breast ultrasonography (**Figures 1A, B**) showed multiple substantial lesions with multiple strong echo masses, categorized as BI-RADS 4c, in the right breast. This score is highly suggestive of cancer under the case presentation section given above. Mammogram of the upper outer quadrant of the right breast showed a lobulated mass with irregular calcification on the craniocaudal view (**Figure 2A**) and mediolateral oblique view (**Figure 2B**). CT scan showed that the edges of the mass remained relatively clear, with uneven density and visible septal changes. Small round foci of fat density were visible on the inner side (**Figures 3A, B**). T1-weighted breast MRI images (**Figure 4A**) showed mixed signal changes in the lesion. The T2-weighted image (**Figure 4B**) exhibited a “fish-meat-like” alteration typical of sarcoma. The solid component on diffusion-weighted imaging (**Figure 4C**) presented high signal intensity, while the apparent diffusion coefficient (ADC) map (**Figure 4D**) showed low signal intensity. Enhanced MRI (**Figure 4E**) demonstrated remarkable enhancement in the solid component compared with T1-weighted images (**Figure 4A**), with no enhancement in the liquefied necrotic area. Numerous large feeding arteries were observed in the periphery (**Figure 4F**). Whole-body bone SPECT showed intense uptake within the right breast, unlike the low or absent uptake seen in breast cancer (**Figures 5A, B**). Pathological section display: Heterotypic osteosarcoma cells can be seen in the bone-like matrix (**Figures 6A–D**), instead of epithelial cells of the opposite sex (**Figures 6A–C**). Immunohistochemical expression is derived from mesenchymal tissue rather than epithelial tissue (**Figure 6D**).

## 3 Discussion

Primary breast osteosarcoma is a rare tumor that mainly originates from the breast stroma (7). Although the exact pathogenesis remains unclear, reports have suggested an association with factors such as radiation exposure, trauma, foreign bodies, and postoperative development from benign tumors (8, 9), which was not present in our case. Unlike osteosarcomas of long bones, primary breast osteosarcoma often occurs in elderly patients (aged 60–80 years) (5), although cases have also been reported in male breasts (10) and even in a 16-year-old female. The literature suggests that the growth of primary breast osteosarcoma is relatively rapid, with an average diameter of about 4.6 cm (4) ADDIN. In contrast, the case reported in this study is of a relatively younger female of 44 years with a large lesion measuring approximately 7.5 cm in diameter.

The diagnosis of primary breast osteosarcoma relies on imaging studies and pathological findings. The most commonly used imaging examinations for breast masses are breast ultrasound (B-ultrasound) and mammography. In this study, both the modalities categorized the lesion as BI-RADS 4C. However, the calcifications within the lesion did not resemble the typical clustered or granular microcalcifications seen in breast cancer. Additionally, there were no classic spiculated margins commonly associated with breast cancer (9) ADDIN. Instead, the calcifications shown on digital radiography (DR) and CT were nodular, irregular, and coarse, similar to the “sunburst” and “transitional zone” patterns described in the literature (11) ADDIN. The largest cross-sectional size of the calcifications was approximately 1.1 cm × 0.8 cm. Nevertheless, some reports suggest that B-ultrasound and mammography cannot distinguish between the benign and malignant nature of breast tumors (5). In our case, the CT and MRI images showed shallow lobulated changes at the edges of the lesion, with locally smooth margins and no spiculation. CT images revealed finer structures within the lesion, with uneven density and small circular areas of fat density. Aribas et al.’s report also shows that CT is helpful to define axillary metastasis, the fixation and invasion of the deep metastatic lesions to the chest wall, which is unique feature of this metastatic tumor. CT could be used for early stage breast metastasis to some extent, but CT was diagnostic in late stage (12). Whole-body bone SPECT showed intense uptake in the local area of the right breast, supporting a diagnosis of sarcoma rather than epithelial-derived carcinoma (4). Primary breast osteosarcoma spreads through the bloodstream rather than lymphatic metastasis (13), which could explain the absence of lymph node metastasis in our case, as observed in DR, CT, MRI, and whole-body SPECT/CT. Primary osteosarcoma of the breast is a rare disease. It is difficult to differentiate it from breast cancer, and the two treatments are different. It is very important not to neglect the diagnosis in the event of the appearance of breast nodules, especially in women in the pre- and post-menopausal period. It is important in all these cases to obtain complete radiological imaging, as was the case with the patient described in the article.

When imaging diagnosis is difficult, pathological biopsy can be used to confirm the diagnosis, It is the gold standard for diagnosis I emphasize the importance of centralizing clinical cases in breast units, conducting multidisciplinary meetings, and that histopathological confirmation is mandatory in all growing masses, even if apparently with unsuspicious clinical–radiological characteristics.

As the pathogenesis of primary breast osteosarcoma remains poorly understood, there is currently no established standardized treatment approach. The 5-year survival rate for primary breast osteosarcoma is relatively low, at about 38% (13)ADDIN. Some experts suggest that treatment should follow the protocols established for sarcomas in other parts of the body, whether it is breast osteosarcoma or other extraskeletal osteosarcomas (9). Given that primary breast osteosarcoma spreads through the bloodstream, extensive surgical resection is essential (6)ADDIN. Some studies indicate that adjuvant chemotherapy may also be helpful (14).

## 4 Conclusions

Primary breast osteosarcoma manifests differently during imaging studies compared with breast cancer. DR, B-ultrasound, and CT show distinct patterns of calcifications, while CT and MRI reveal differences in margins, density, and signal intensity. Whole-body SPECT can assist in differentiation based on tracer uptake. Pathology can distinguish between two types. So for breast tumors we should emphasize even more the importance of preoperative puncture biopsy.

## Data Availability

The original contributions presented in the study are included in the article/supplementary material. Further inquiries can be directed to the corresponding author/s.
